# Identification and validation of critical genes with prognostic value in gastric cancer

**DOI:** 10.3389/fcell.2022.1072062

**Published:** 2022-12-14

**Authors:** Ningxin Dong, Xiaolong Ma, Jing Shen, Yunlu Zheng, Guiyuan Li, Shaoqiang Zheng, Xiaoyi Huang

**Affiliations:** ^1^ Department of Radiology, Tongji Hospital, School of Medicine, Tongji University, Shanghai, China; ^2^ Department of Information, Tongji Hospital, School of Medicine, Tongji University, Shanghai, China; ^3^ Department of Oncology, Tongji Hospital, School of Medicine, Tongji University, Shanghai, China; ^4^ Department of Neonatology, International Peace Maternity and Child Health Hospital, School of Medicine, Shanghai Jiao Tong University, Shanghai, China; ^5^ Shanghai Key Laboratory of Embryo Original Diseases, Shanghai, China; ^6^ Shanghai Municipal Key Clinical Speciality, Shanghai, China

**Keywords:** gastric cancer, prognostic model, MYL9, epithelial-mesenchymal transition, metastasis

## Abstract

**Background:** Gastric cancer (GC) is a digestive system tumor with high morbidity and mortality rates. Molecular targeted therapies, including those targeting human epidermal factor receptor 2 (HER2), have proven to be effective in clinical treatment. However, better identification and description of tumor-promoting genes in GC is still necessary for antitumor therapy.

**Methods:** Gene expression and clinical data of GC patients were downloaded from The Cancer Genome Atlas (TCGA) and Gene Expression Omnibus (GEO) databases. Last absolute shrinkage and selection operator (LASSO) Cox regression were applied to build a prognostic model, the Prognosis Score. Functional enrichment and single-sample gene set enrichment analysis (ssGSEA) were used to explore potential mechanisms. Western blotting, RNA interference, cell migration, and wound healing assays were used to detect the expression and function of myosin light chain 9 (MYL9) in GC.

**Results:** A four-gene prognostic model was constructed and GC patients from TCGA and meta-GEO cohorts were stratified into high-prognosis score groups or low-prognosis score groups. GC patients in the high-prognosis score group had significantly poorer overall survival (OS) than those in the low-prognosis score groups. The GC prognostic model was formulated as PrognosisScore = (0.06 × expression of BGN) - (0.008 × expression of ATP4A) + (0.12 × expression of MYL9) - (0.01 × expression of ALDH3A1). The prognosis score was identified as an independent predictor of OS. High expression of MYL9, the highest weighted gene in the prognosis score, was correlated with worse clinical outcomes. Functional analysis revealed that MYL9 is mainly associated with the biological function of epithelial-mesenchymal transition (EMT). Knockdown of MYL9 expression inhibits migration of GC cells *in vitro*.

**Conclusion:** We found that PrognosisScore is potential reliable prognostic marker and verified that MYL9 promotes the migration and metastasis of GC cells.

## Introduction

Gastric cancer (GC) is the fourth leading cause of cancer worldwide (Sung et al., 2021). With improvements in public health consciousness, some patients are being diagnosed with early stage GC during gastric endoscopy, resulting in prolonged survival (Lee et al., 2021). However, numerous patients still have a poor prognosis due to advanced-stage diagnosis with metastasis, which is an important contributor to cancer recurrence and complications (Lee et al., 2021). Therefore, it is essential to elucidate the mechanisms underlying GC metastasis.

Epithelial-mesenchymal transition (EMT) refers to the process by which cells lose their epithelial characteristics and acquire mesenchymal features (Bakir et al., 2020). As cells undergo EMT, intercellular junctions and connections diminish and cell contractility and motility increase, which facilitates cell adherence and penetration into vessels for further metastasis. The conversion of epithelial cells to mesenchymal cells is a continuous process (Bakir et al., 2020). In tumors, multitudinous cells display multiple degrees of epithelial and mesenchymal properties, as well as differences in cellular invasiveness and metastatic potential (Bakir et al., 2020). As EMT can crosstalk with other important malignant processes such as stemness and vascularization (Pastushenko and Blanpain, 2019), targeting critical nodes in the EMT network can be a potential therapeutic strategy.

Myosin is a hexameric protein that contains two heavy chains, two alkali light chains, and two regulatory light chains (Jiang et al., 2014). The myosin light chain 9 (MYL9)-encoded protein is one of the two regulatory light chains that binds calcium and is activated by myosin light chain kinase. The myosin regulatory subunit plays an important role in both smooth muscle and non-muscle cell contractile activities (Jiang et al., 2014). MYL9 participates in vessel formation by activating actomyosin contractility and angiogenic sprouting (Abraham et al., 2009). In addition, MYL9 participates in local blood vessel impairment to support nutrition leaks (Meng et al., 2021). MYL9 also participates in cell proliferation because it affects the contractile ring formation of mitotic cells during cytokinesis (Jiang et al., 2014). Studies have also found that YAP remodels the extracellular matrix and promotes cancer cell migration by regulating MYL9 and the actomyosin cytoskeleton in cancer-associated fibroblasts (Calvo et al., 2013). As cell contractility and motility increase during metastasis, whether MYL9 affects cancer metastasis directly *via* cellular motility enhancement and EMT status changes remains unclear.

In this study, we established an evaluation model with four indicators to estimate GC prognosis. In this model, MYL9 had the highest ratio and was selected for functional verification. We further demonstrated that MYL9 deficiency inhibited cancer cell migration using scratch and transwell assays. Thus, our model provides an efficient method for identifying novel therapeutic targets.

## Methods

### Gastric cancer data sets and preprocessing

The microarray data displayed in Table 1 were downloaded from Gene Expression Omnibus (GEO; https://www.ncbi.nlm.nih.gov/geo) and The Cancer Genome Atlas (TCGA). To avoid errors caused by different sequencing platforms, we chose the GEO dataset from the same platform (Affymetrix Human Genome U133 Plus 2.0 Array). Furthermore, gene expression data (FPKM normalized, reads per kilobase of exon model per million mapped reads) and the corresponding clinical datasets for GC tissue samples and adjacent normal tissue samples of 417 patients in TCGA were downloaded from UCSC Xena (https://xenabrowser.net/datapages/).

**TABLE 1:** Basic information regarding the series used in the study.

Series accession numbers	Platform used	Platform	No. of input patients	Region
TCGA-STAD	Illumina RNAseq	HiSeq 2000	417	United States
GSE64951	Affymetrix Human Genome U133 Plus 2.0 Array	GPL570	94	United States
GSE54129	Affymetrix Human Genome U133 Plus 2.0 Array	GPL570	132	China
GSE29272	Affymetrix Human Genome U133 Plus 2.0 Array	GPL570	268	United States
GSE13911	Affymetrix Human Genome U133 Plus 2.0 Array	GPL570	69	Italy
ACRG	Affymetrix Human Genome U133 Plus 2.0 Array	GPL570	300	Asia

Raw microarray data from Affymetrix^®^ were downloaded and normalized using a robust multi-array averaging method. Affy and simpleaffy packages were used to normalize the Affymetrix data. For gene expression profiles of platforms other than Affymetrix, normalized matrix files were downloaded directly. Probe signals corresponding to the same transcript were aggregated using the standard probe assignment method (hgu133plus2cdf) and normalized as log2 (expression+0.1) of the transcripts for the genes.

### Calculating differentially expressed genes

The gene expression differences (DEGs) between the normal and tumor groups in TCGA-STAD were compared using the limma (Law et al., 2014) package in R software, and the DEGs were identified with the cutoff logFC ≥2, logFC < -2, and *p*-value < 0.05. The Sva:ComBat algorithm was used to remove batch effects between the four GEO datasets. The ComBat method (Johnson et al., 2007) is based on the empirical Bayes approach. Surrogate variable analysis (SVA) (Leek and Storey, 2007) has been widely used to estimate hidden covariates (technical and biological). After the batch effect was removed, the DEGs between the normal and tumor groups in the GEO cohorts were also calculated using the limma package, and the DEGs were identified with the cutoff of logFC ≥1 and logFC < -1, and *p*-value < 0.05.

### Prognostic model establishment

The gene expression differences between the normal and tumor groups were compared using limma in the TCGA-STAD and GEO data cohorts, respectively. The intersection of these two genes was used to obtain the final DEGs. The final DEGs were selected to build a prognosis score model.

In the TCGA-STAD data queue, the samples were randomly divided into training and testing sets in a ratio of 7:3. LASSO Cox was used to build the prognosis score model. The PrognosisScore model was constructed based on the fraction of selected genes using Cox regression coefficients. The function for the prognosis score is:

$$PrognosisScore=\sum_{i=1}^{n}\left(\beta_{i*}Exp_{j}\right)$$

where Exp is the selected gene, and *β* is the coefficient of the selected gene.

The timeROC package (Heagerty et al., 2000) was used to plot and visualize the receiver operating characteristic (ROC) curves. The area under the ROC curve (AUC) and confidence intervals were quantified using the R package time ROC. The procedure was conducted in the above testing set, which was left before the model training of the prognosis score.

### Cell culture

The GC cell lines FU97, AGS, and NCI-N87 used in our study were purchased from the American Type Culture Collection (ATCC), and HS-746T, MGC803, MGC823, SGC7901, MKN45, MKN28, HGC27, and GES-1 cell lines were purchased from the Shanghai Institutes for Biological Sciences, Chinese Academy of Sciences. All GC cell lines were authenticated by short tandem repeat analysis and were negative for *mycoplasma*. HS-746T, AGS, NCI-N87, MGC823, SGC7901, MKN45, MKN28, and HGC27 cells were cultured at 37°C in a humidified atmosphere of 5% CO_2_ in RPMI-1640 medium (Gibco, San Francisco, CA, United States) containing 10% fetal bovine serum (Gibco), 100 U/mL penicillin, and 100 U/mL streptomycin (Sangon Biotech, Shanghai, China). GES-1, FU97, and MGC803 cells were cultured at 37°C in a humidified atmosphere of 5% CO_2_ in Dulbecco’s modified Eagle’s medium (DMEM; Gibco) containing 10% fetal bovine serum, 100 U/mL penicillin, and 100 U/mL streptomycin.

### Cell migration assay

For the migration assay, the cells were suspended in serum-free medium (1 × 10^5^ cells) and added to the upper chamber of a 24-well insert (membrane pore size, 8 mm; Corning Life Sciences, MA, United States). Medium containing 10% serum was added to the lower chamber. After incubation for 24 h, cells that migrated to the bottom of the membranes were fixed and stained with 0.1% crystal violet for 30 min.

### Wound healing assay

Briefly, transfected cells were seeded into 6-well plates at a concentration of 5 × 10^5^ cells/ml. When cells were cultured to approximately 90% confluency, a 200 μl pipette tip was used to make a straight wound on the confluent monolayer. The cells were then cultured in serum-free medium for 48 h. Wounded monolayers were washed with phosphate-buffered saline (PBS) and photographed using an inverted microscope.

### Western blot

Cells were lysed using RIPA lysis buffer (Solarbio, Beijing, China) containing a proteinase inhibitor cocktail (Sigma, St. Louis, MO, United States). A total of 20 mg of protein was used for each western blot analysis. Proteins were subjected to vertical electrophoresis and transferred to a polyvinylidene fluoride (PVDF) membrane. The membrane was incubated with anti-MYL9 (31244, Signalway Antibody) and anti-GAPDH (ab8245, Abcam) antibodies, followed by incubation with secondary antibodies. The bands were detected using a Tanon enhanced chemiluminescence (ECL) imaging system.

### RNA interference

Small interfering RNAs (siRNAs) targeting MYL9 were purchased from GenePharma (Shanghai, China). The siRNA sequences of MYL9 were (5′-AGG​AAG​UGG​ACG​AGA​UGU​ACC-3′) and (5′-CAA​UGU​CUU​CGC​AAU​GUU​UGA-3′). The human GC cell lines MGC803 and MKN28 were transfected with MYL9 siRNA using Lipo3000 (Thermo Fisher Scientific, United States). The detailed procedure was performed in accordance with the manufacturer’s instructions.

### Statistical analysis

Statistical analyses were performed using R software (version 4.1.3). Kaplan-Meier survival analysis was employed with overall survival (OS) as the outcome metric, and the log-rank test or hazard ratio test was used to determine the statistical significance of differences. Statistical significance was set at *p* < 0.05.

## Results

**FIGURE 1:**
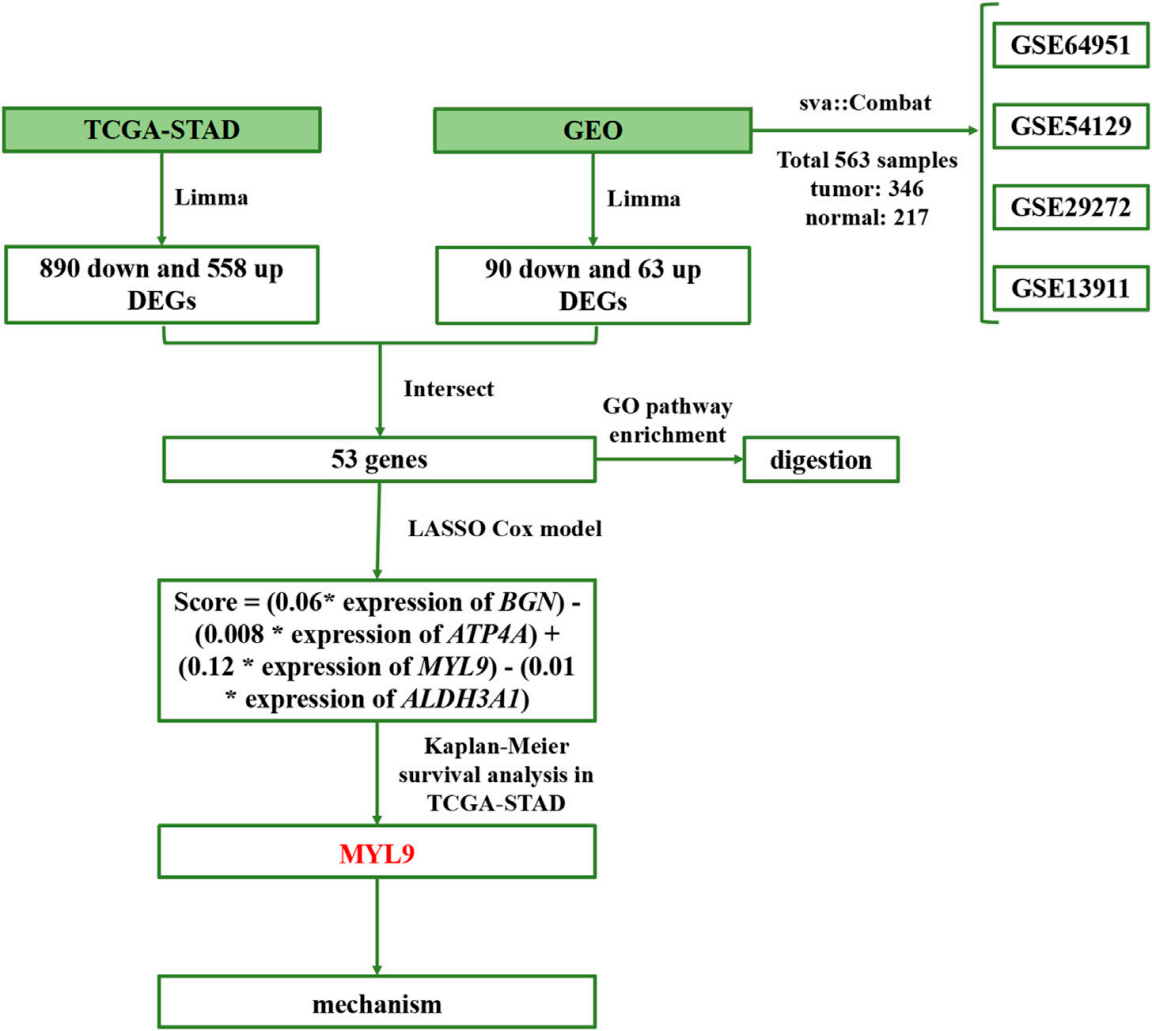
Flowchart of the study.

In this study, we used four main steps to establish an accurate and reliable prognostic signature for GC (Figure 1): identification of differentially expressed genes (DEGs), establishment of prognostic models, determination of survival mechanisms, and experimental verification.

### Identification of differentially expressed genes

**FIGURE 2:**
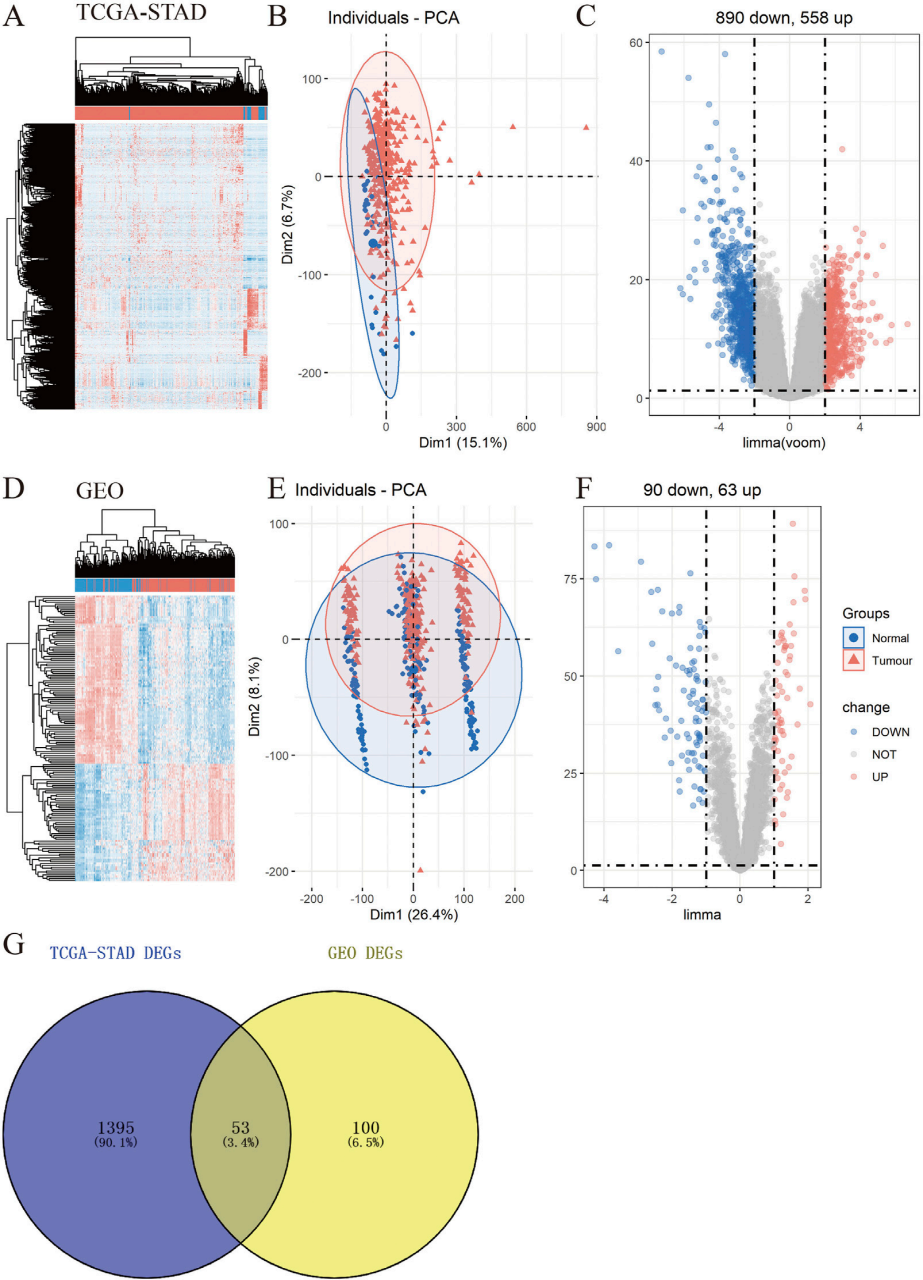
Identification of differentially expressed genes (DEGs) in TCGA-STAD and GEO data cohorts. **(A–F)** Heatmap, PCA, and Volcano plot of DEGs between normal and tumor samples in TCGA-STAD **(A–C)** and GEO **(D–F) (G)** Venn diagram between TCGA-STAD DEGs and GEO DEGs.

To explore the underlying molecular functions of GC, DEGs were calculated using the TCGA-STAD and GEO databases. Specifically, in TCGA-STAD data cohort, 1,448 genes in tumor samples presented significant differences in expression compared to normal samples (*p* < 0.05, |logFC|≥2), 890 genes were markedly downregulated, and 558 genes were upregulated (Figures 2A–C). For the four GEO data cohorts, we used the ComBat algorithm of the sva R package to correct the batch effects from non-biological technical biases. From the PCA and boxplot diagrams, it was found that the batch effect of the four datasets was well removed (Supplementary Figures S1A–D). Based on the 217 normal samples and 346 tumor samples, 153 genes in tumor samples presented significant differences in expression compared to normal samples (*p* < 0.05 and |logFC|≥1), 90 genes were markedly downregulated, and 63 genes were upregulated (Figures 2D–F). The DEGs obtained from TCGA-STAD and GEO were intersected to obtain 53 DEGs (Figure 2G).

**FIGURE 3:**
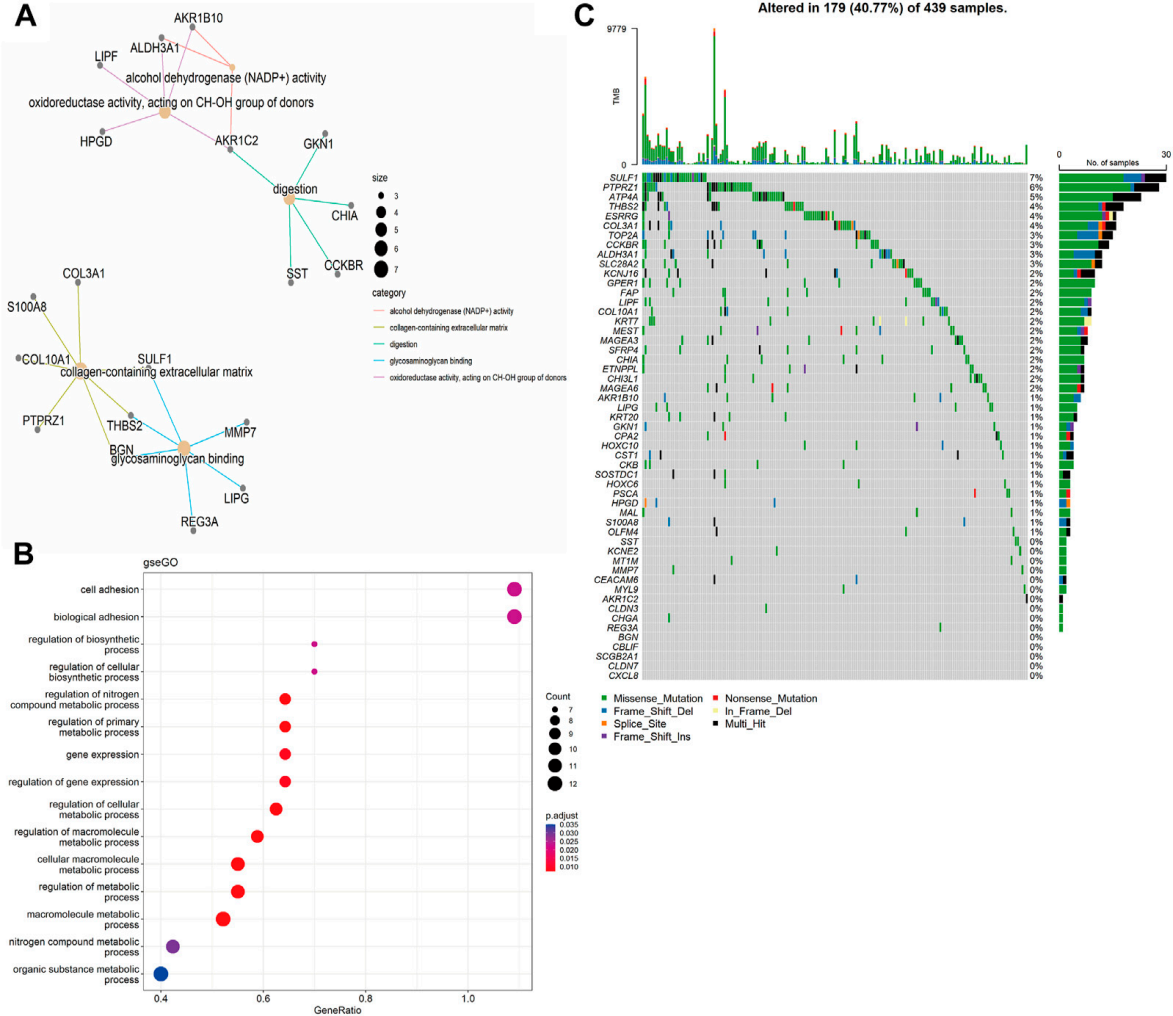
Enrichment analysis. **(A)** Gene Ontology (GO)-tree analysis; **(B)** gesGO pathway enrichment analysis **(C)** The mutation landscape of key DEGs in TCGA-STAD cohort.

Functional annotations of Gene Ontology (GO) and Kyoto Encyclopedia of Genes and Genomes (KEGG) enrichment indicated that the DEGs obtained from TCGA-STAD were enriched in digestion and matrix remodeling related pathways, like cell adhesion, protein digestion and absorption, PPAR signaling, extracellular matrix organization, extracellular structure organization, and so on (Supplementary Figures S2A,B). Meanwhile, the DEGs obtained from GEO were also enriched in digestion and matrix remodeling related pathways, such as protein digestion and absorption, focal adhesion, extracellular matrix organization, extracellular structure organization, wound healing, and so on (Supplementary Figures S2C,D). And these intersected genes were significantly associated with digestion, collagen-containing extracellular matrix, cell adhesion, and biological adhesion (Figures 3A,B). Furthermore, SULF1, PTPRZ1, ATP4A, THBS2, ESRRG, COL3A1, TOP2A, CCKBR, ALDH3A1, and SLC28A2 had high missense mutation rates (Figure 3C).

### Establishment of PrognosisScore model

**FIGURE 4:**
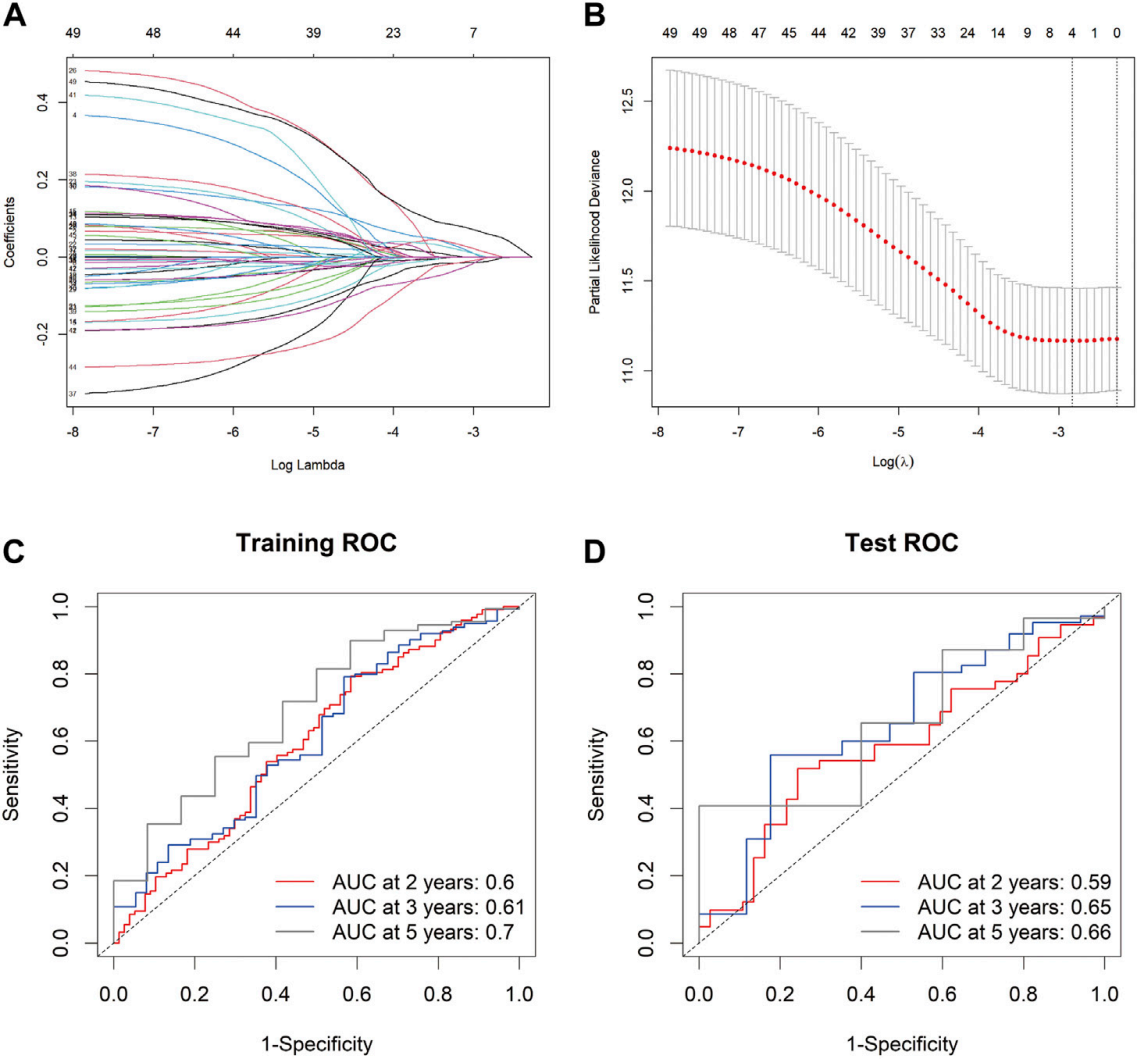
The PrognosisScore model and its prognostic significance. **(A)** Least absolute shrinkage and selection operator (LASSO) coefficient profiles of the four key molecules. **(B)** Tuning parameter selection by tenfold cross-validation in the LASSO model. The partial likelihood deviance was plotted against log (Lambda/λ), and λ was the tuning parameter. The partial likelihood deviance values are shown, and error bars represented s. e. The dotted vertical lines showing the optimal values through minimum criteria and 1 -s.e. Criteria. **(C,D)** The PrognosisScore model was measured by time-dependent receiver operating characteristic (ROC) curves in the training set and the test set.

To develop a gene expression-based prognostic signature for GC, the gene expression differences between GC tissues and adjacent normal tissues in TCGA-STAD and GEO data cohorts were compared using the limma package. The intersection of these two genes was used to obtain the final DEGs. The final DEGs were selected to build a prognosis score model. Since TCGA has comprehensive clinical information, we built the prognosis model in the TCGA dataset. In the TCGA-STAD data queue, the samples were randomly divided into training and testing sets in a ratio of 7:3. LASSO Cox was used to build the prognosis score model (Figures 4A,B). A risk score was calculated for each patient using a formula derived from the expression levels of the four genes weighted by their regression coefficient: PrognosisScore = (0.06 * expression of BGN) - (0.008 * expression of ATP4A) + (0.12 * expression of MYL9) - (0.01 * expression of ALDH3A1).

**FIGURE 5:**
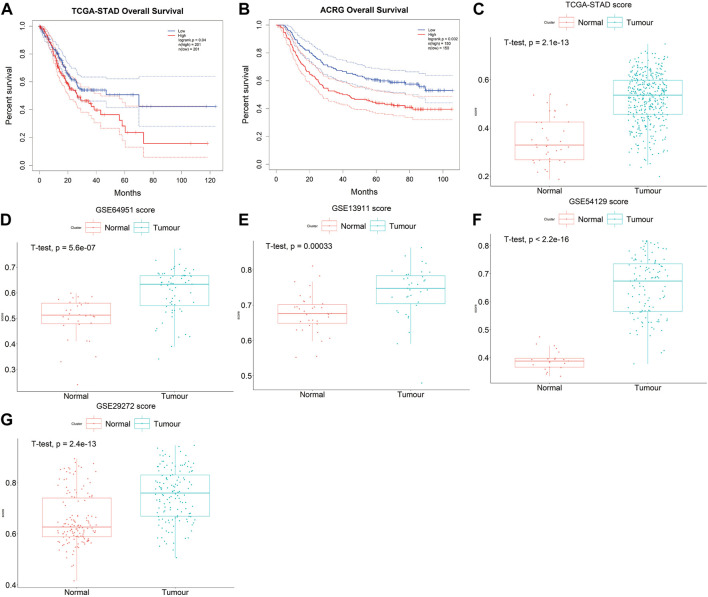
The PrognosisScore mode has strong robustness in predicting prognosis. **(A,B)** Survival analyses for low- and high-PrognosisScore groups using Kaplan-Meier curves (Log-rank test) in **(A)** TCGA-STAD and **(B)** ACRG data cohorts; **(C–G)** The boxplot of PrognosisScore in tumor and normal group. **(C)** TCGA-STAD, **(D)** GSE64951 (ACRG), **(E)** GSE13911 **(F)** GSE54129, and **(G)** GSE29272. The thick line represented the median value. The bottom and top of the boxes are the 25th and 75th percentiles (interquartile range), respectively.

The prognostic accuracy of the prognosis score, assessed as a continuous variable, was investigated using a time-dependent ROC analysis. The average AUC values of the 2-, 3-, and 5-year prognosis predictions for the training set reached 0.6, 0.61, and 0.70, respectively. For the prediction on the test set, the average AUC values of 2-, 3-, and 5-year survival were 0.59, 0.65, and 0.66, respectively (Figures 4C,D). Moreover, the samples were classified into PrognosisScore_High and PrognosisScore_Low groups according to the median value. Kaplan-Meier survival analyses (Figures 5A,B) showed that the PrognosisScore_High group had poorer OS than the PrognosisScore_Low group. Furthermore, the mean prognosis score was higher in the tumor group than that in the normal group in both the TCGA-STAD and GEO data cohorts (Figures 5C–G).

Quite encouragingly, the PrognosisScore could accurately discriminate the survival of patients within the same TNM stages. Whether in the early or late stages of TNM stages (Pathologic_T:Tumor, Pathologic_N:Node, Pathologic_M:Metastasis), the PrognosisScore can accurately discriminate the survival of GC patients within the same stages, except M1 stage, as the sample number of M1 stage is too small (less than 30), it is not suitable to use KM curve to evaluate prognosis (Supplementary Figures S3A–F).

### The correlation of MYL9 and EMT

**FIGURE 6:**
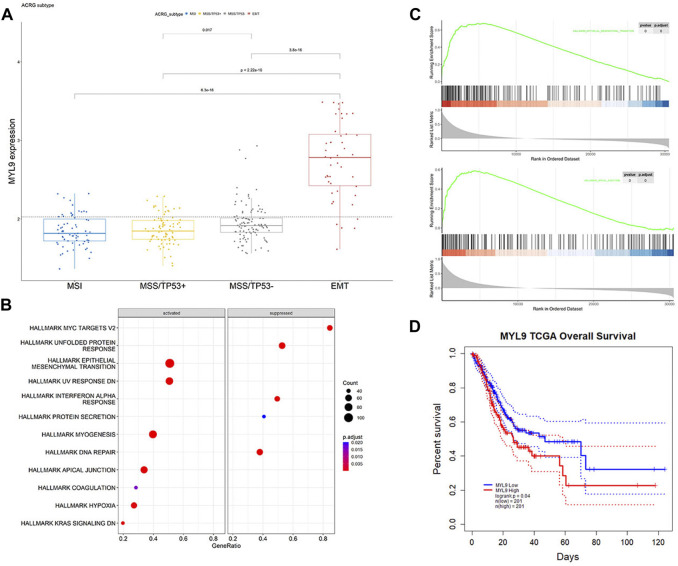
MYL9 is positively correlated with EMT. **(A)**The expression of MYL9 in four different stages (MSI, MSS/TP53^+^, MSS/TP53^-^, EMT) of ACRG data cohort. The thick line represents the median value. The bottom and top of the boxes are the 25th and 75th percentiles (interquartile range), respectively. **(B)** Gene set enrichment analysis (GSEA) of MYL9; **(C)**GSEA revealed significantly activated signaling pathways of MYL9 **(D)**Survival impact of the MYL9 expression, Kaplan-Meier curves for overall survival (OS) in TCGA-STAD cohort.

In PrognosisScore model, ATP4A and ALDH3A1 with negative regression coefficients were low expressed in GC (Supplementary Figures S4A,B), while BGN with positive regression coefficients were high expressed in GC (Supplementary Figure S4C). Furthermore, in ACRG cohort, the average expression of MYL9 was significantly higher in the EMT subgroup of GC patients (Cristescu et al., 2015) (Figure 6A). MYL9 is a typical intracellular myosin subunit that plays an important role in morphology, growth, and migration of epithelial cells. All transcripts were quantified and ranked by log2 (fold change) between MYL9-High and MYL9-Low expression groups (group by median), and Gene set enrichment analysis (GSEA) was performed with all transcripts using R packages GSVA and clusterProfiler. We found gene sets related to the EMT pathway and apical junction enriched in the MYL9-High expression group (Figures 6B,C). We assessed the correlation between MYL9 expression and survival of patients with GC. Kaplan–Meier Plotter analysis showed that high MYL9 expression was associated with poor clinical outcomes in GC patients (Figure 6D).

### Experimental verification of MYL9 deficiency inhibiting GC cell migration *in vitro*

**FIGURE 7:**
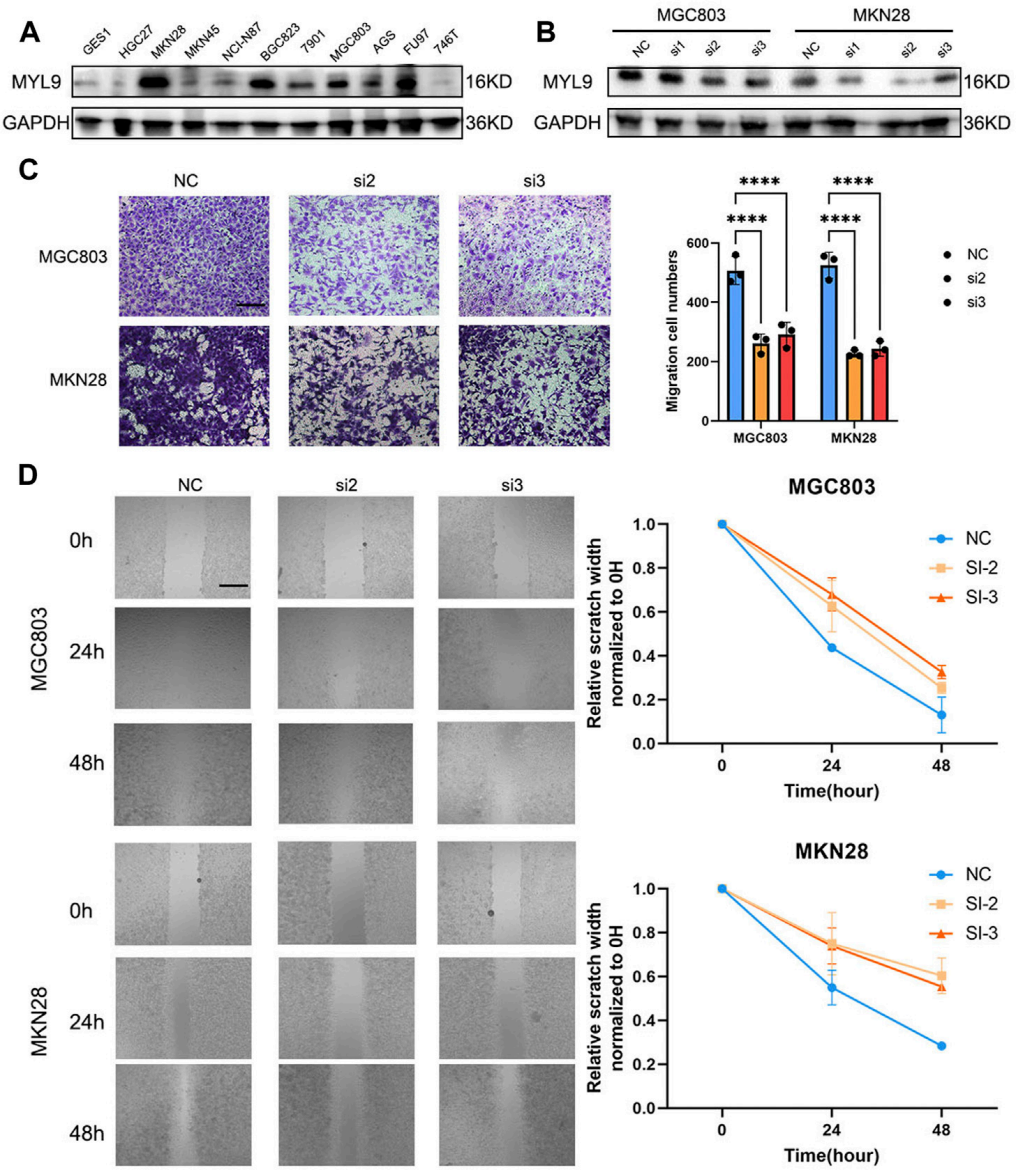
MYL9 affected GC cell migration. **(A)** The MYL9 expression in 10 GC cell lines and one immortalized stomach epithelial cell line. **(B)** Knockdown of MYL9 by siRNA in MKN28 and MGC803 cells. **(C)** The migration ability measured by transwell migration assays. (Scale bar: 250 μm) **(D)** Migration ability. The statistical analysis is shown in the bar graphs. (Scale bar: 250 μm).

To determine the biological function of MYL9, we first examined the basal expression of MYL9 in 10 GC cell lines and GES1 (Figure 7A), and we constructed MYL9 knockdown cells (MGC803-siMYL9 and MKN28-siMYL9) by transfecting GC cells with small-interfering RNAs. siRNA-2 and siRNA-3 inhibited MYL9 expression more efficiently and were chosen for subsequent experiments (Figure 7B). To further examine the effect of aberrant MYL9 expression on GC cell migration potential, we performed transwell assays, and MYL9 knockdown decreased the number of cells that had migrated to the lower compartment in MGC803 and MKN28 cells (Figure 7C). Wound healing assays were also performed to evaluate cell migratory ability, and the results revealed that MYL9 knockdown reduced the velocity of scratch healing in both two designed siRNAs in MGC803 and MKN28 cells (Figure 7D). Our results showed that MYL9 expression could alter cell migratory ability, which might affect the tumor metastatic potential.

## Discussion

GC poses a heavy health and economic burden to patients and is the fifth most common cancer worldwide (Sung et al., 2021). Identifying efficient treatments for patients with distant metastasis or recurrence remains an intractable challenge (Lee et al., 2021). Effective signatures will largely benefit prognosis prediction and therapeutic decisions, serving as an indispensable part of precision medicine (Khambata-Ford et al., 2007; Salazar et al., 2011; Chen et al., 2016; Sparano et al., 2019). Many cancer biomarkers have been elucidated, such as KRAS in colorectal cancer (Dienstmann et al., 2015), human epidermal factor receptor 2(HER2) in breast cancer (Harbeck and Gnant, 2017), and EGFR in lung cancer (Paez et al., 2004). However, for patients with GC, there are few therapeutic indicators, and further exploration is required. In our study, we integrated the genomic profiling of 254 normal gastric samples and 726 GC samples to explore the key molecules that mediate patient prognosis. We found that genomic differences between normal and tumor tissues were significantly correlated with EMT-related pathways. We first calculated significant DEGs in tumors compared to normal samples based on a large population in TCGA-STAD and GEO databases to unveil novel potential markers. We used the LASSO Cox regression model to construct the PrognosisScore to integrate the significant roles of the DEGs. The GC prognostic model was formulated as PrognosisScore = (0.06 × expression of BGN) - (0.008 × expression of ATP4A) + (0.12 × expression of MYL9) - (0.01 × expression of ALDH3A1). From the KM curve, ALDH3A1, ATP4A and BGN were not significant for prognosis, whereas the expression level of MYL9 was negatively correlated with favorable outcomes (Supplementary Figures S4D–F, Figure 6D). The prognostic accuracy of the PrognosisScore model was assessed using external TCGA-STAD and GEO data cohorts. Thus, the constructed PrognosisScore signature can serve as a robust and independent method for predicting GC patient outcomes.

Current efforts are focused on predictive accuracy over explanatory power (Cho et al., 2011; Tan et al., 2011; Wang et al., 2011; Cancer Genome Atlas Research, 2014; Cristescu et al., 2015). Indeed, effective gene signatures usually involve the abnormal activation of multiple intracellular signaling pathways and cascade reactions, which will yield promising new candidate therapeutic targets (Cancer Genome Atlas Research, 2014; Cristescu et al., 2015). To explore the underlying molecular rationale, we conducted functional annotations of GO enrichment, which indicated that our gene set was significantly associated with digestion and collagen-containing extracellular matrix.

It is commonly acknowledged that EMT has a tight connection with tumor metastasis (Pastushenko and Blanpain, 2019). With investigations focusing on EMT in cancer, it has been found that EMT also interacts with many cancer functional characteristics, such as tumor initiation, stemness, and resistance to therapy (Pastushenko and Blanpain, 2019), which can be exploited as therapeutic targets based on molecular properties and crosstalk. GO enrichment of our gene sets indicated collagen-containing extracellular matrix, which affected the difficulty of cell migration. BGN and MYL9 were also found to be fibroblast-specific markers of poor prognosis in colorectal cancer (Zhou et al., 2020). In GC, high BGN expression is significantly associated with poor patient survival (Zhang et al., 2022). In addition, BGN was found to drive EMT in pancreatic ductal adenocarcinoma (Thakur et al., 2016). Extracellular matrix remodeling and cancer cell invasion require actomyosin cytoskeleton, and MYL9 is regulated by YAP and participates in matrix stiffening (Calvo et al., 2013; Feng et al., 2022). In breast cancer, MYL9 is activated by myocardin-related transcription factor-A and promotes MCF-7 cell migration (Luo et al., 2014). A recent study also found that MYL9 is a reliable prognostic gene that influences invasion in GC (Wang et al., 2022). Although some bioinformatic studies have found that MYL9 plays a role in cancer migration ability and metastasis, molecular proof is scarce about MYL9 function in GC cells. In our study, we found MYL9-high expression group gene sets related to the EMT pathway and apical junction in GSEA analysis (Figures 6B,C). EMT can endow cells with adhesive, migratory, and invasive abilities during tumor development and progression (Bill and Christofori, 2015; Cao et al., 2018). MYL9 has been demonstrated to affect intracellular myosin contractility and cell motility through various mechanisms during the progression of other disease (Abraham et al., 2009; Jiang et al., 2014; Meng et al., 2021). In this study, we identified MYL9 as an important indicator in a novel model that analyzed and predicted the prognosis of patients with GC. Clinical and pathological analyses showed that MYL9 expression positively correlated with advanced TNM stage, local invasion, lymph node metastasis, and poor prognosis. *In vitro* experiments further revealed that MYL9 significantly promoted the metastatic potential of GC cells. Our findings indicate that our model is efficient in predicting patient prognosis. MYL9 is a novel pro-metastatic gene that could serve as an independent indicator and therapeutic target in patients with metastatic GC.

However, our study has some limitations that should be acknowledged. First, considering that the clinical annotation information available in publicly available datasets was limited, the clinicopathological parameters analyzed in the present study could not be comprehensive, which may have resulted in a potential bias in the predictive performance of the PrognosisScore signature. Second, all the GC transcriptome profiles used for constructing the PrognosisScore were based on the Affymetrix Human Genome U133 Plus 2.0 Array-GPL570 and Illumina RNAseq platforms. Therefore, caution should be exercised when applying the PrognosisScore signature to GC samples tested on other platforms. Further research is required to confirm the specific functional mechanisms of MYL9 in GC. In summary, our research provides important resources for elucidating the specific role of MYL9 in the EMT of GC.

## Data Availability

The datasets presented in this study can be found in online repositories. The names of the repository/repositories and accession number(s) can be found in the article/Supplementary Material.
