# Supplementary figures and images for: Identification and validation of critical genes with prognostic value in gastric cancer
Source: Front Cell Dev Biol. 2022 Dec 14;10:1072062. doi: 10.3389/fcell.2022.1072062 (PMC9795222; doi:10.3389/fcell.2022.1072062)

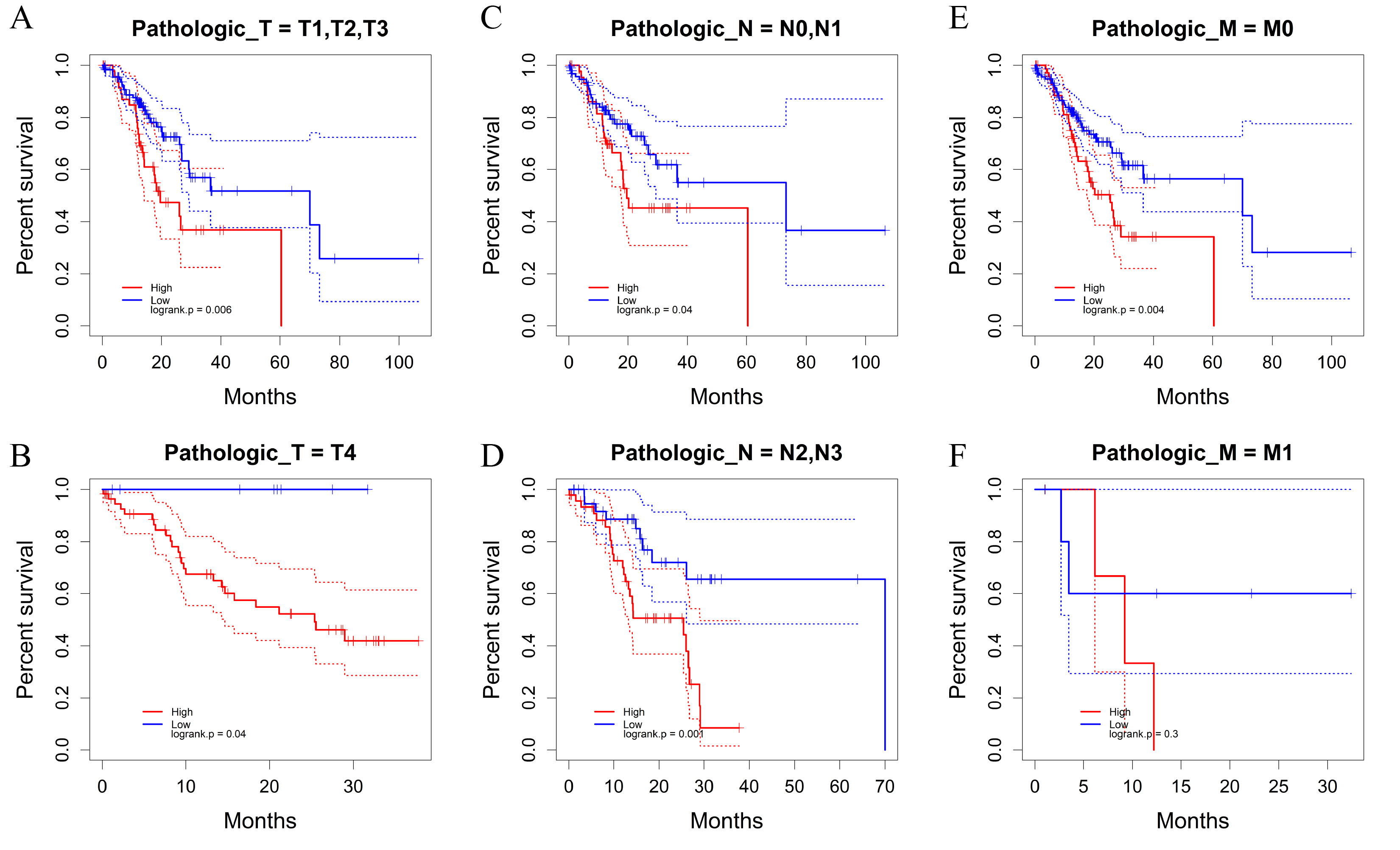

Supplement: Supplementary file 1 [file Image3.JPEG]

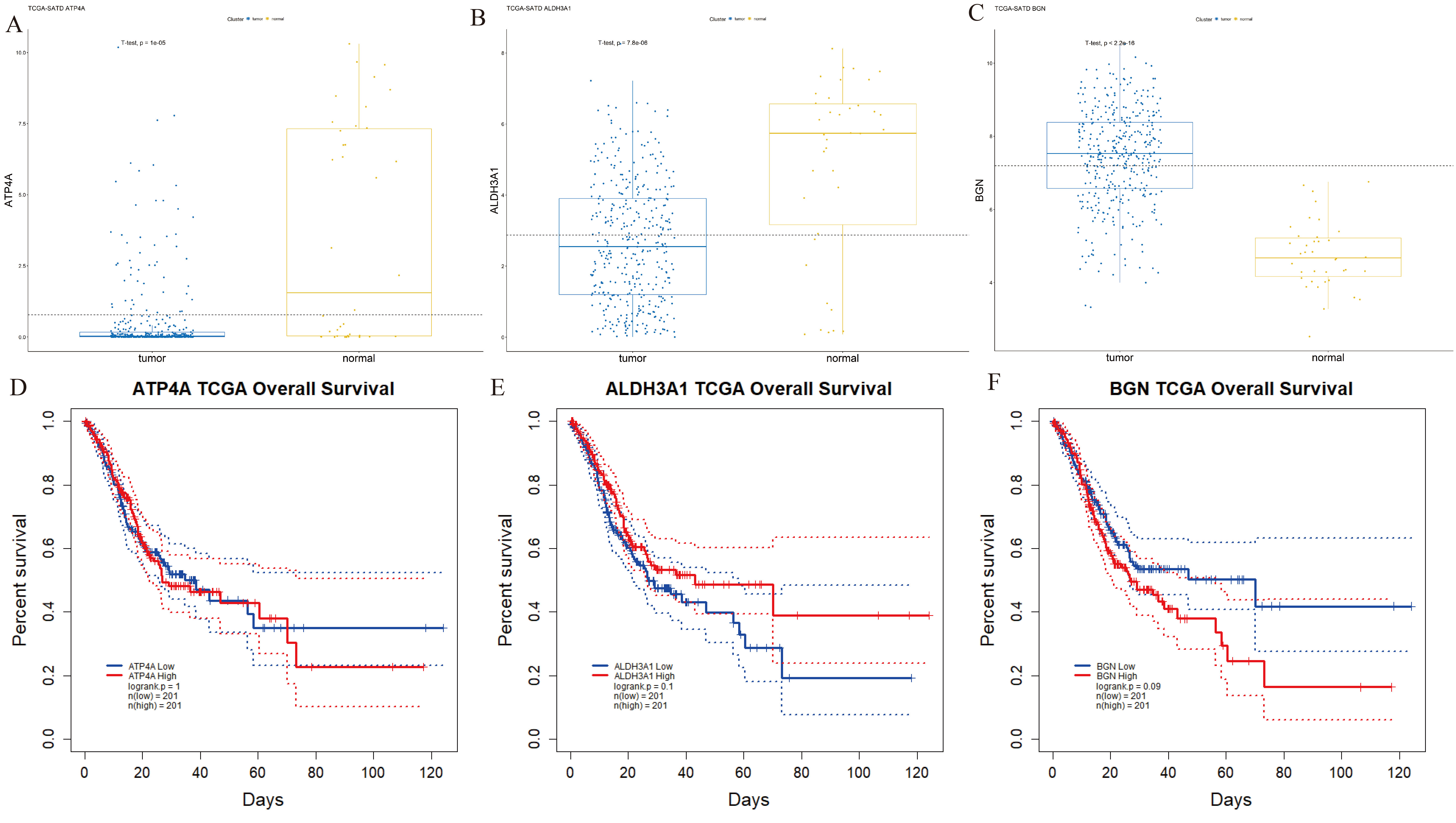

Supplement: Supplementary file 2 [file Image4.JPEG]

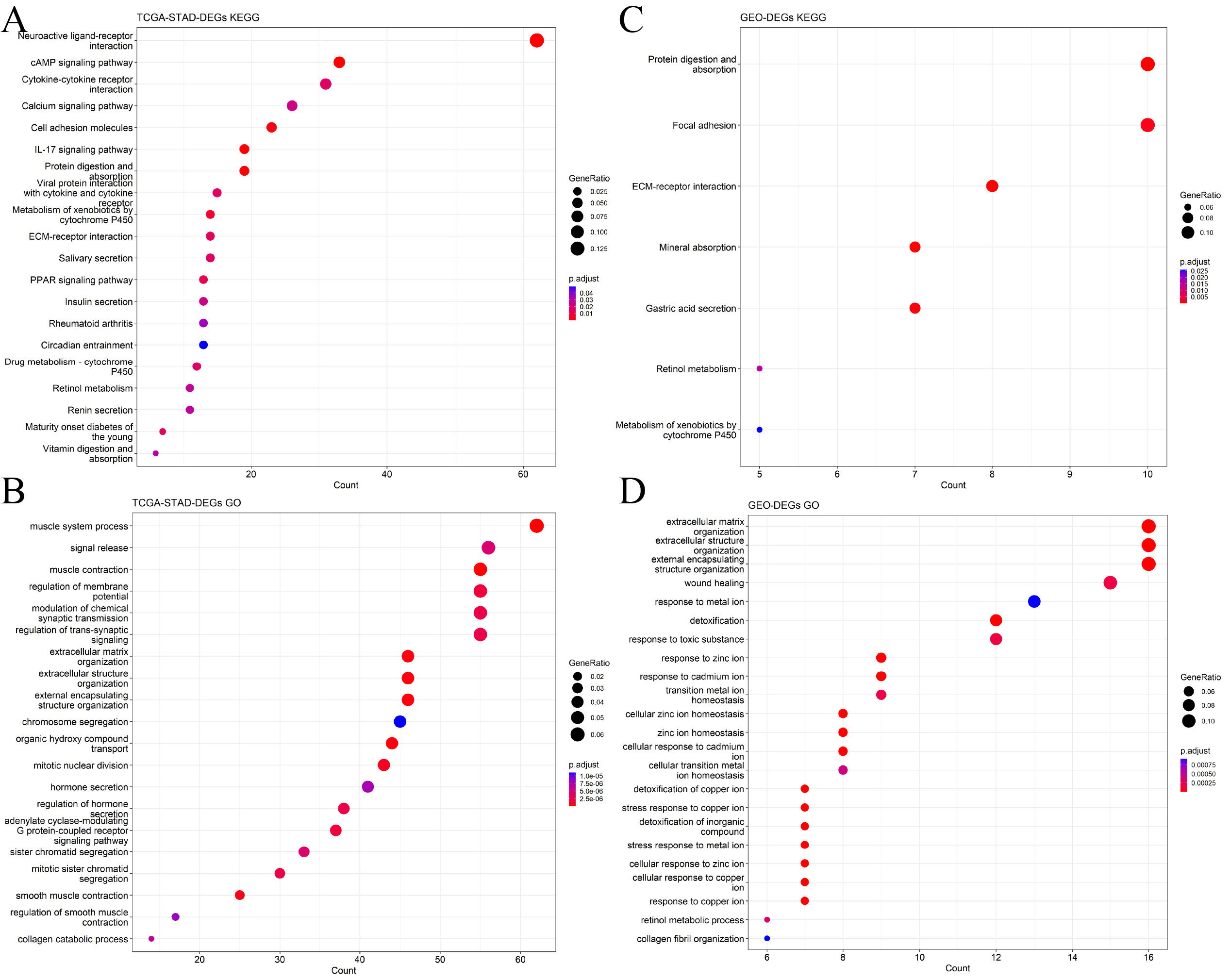

Supplement: Supplementary file 3 [file Image2.JPEG]

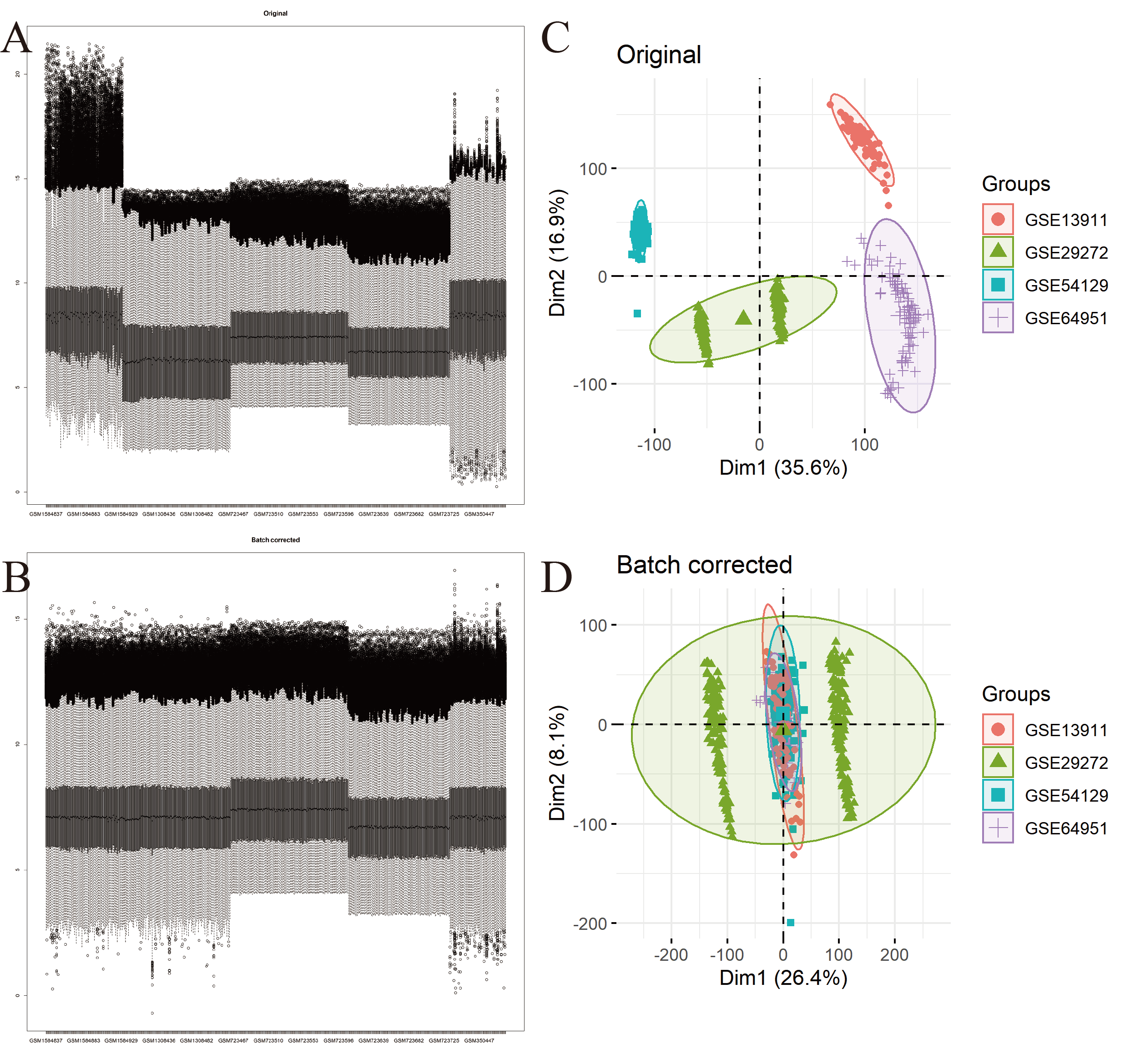

Supplement: Supplementary file 4 [file Image1.TIF]
