# Supplementary material for: Identification and validation of critical genes with prognostic value in gastric cancer
Source: Front Cell Dev Biol. 2022 Dec 14;10:1072062. doi: 10.3389/fcell.2022.1072062 (PMC9795222; doi:10.3389/fcell.2022.1072062)
Supplement: Supplementary file 5 [file DataSheet1.DOCX]

Supplementary Material

Figure S1. Removing the batch effect of four GEO data sets. (A-B) The boxplot (A) before and (B) after batch removal of all samples in four GEO datasets. (C-D) The principal component analysis for all samples in four GEO datasets (C) before and (D) after batch effect removal.

Figure S2. Enrichment analysis. (A-B) Kyoto Encyclopedia of Genes and Genomes (KEGG) and Gene Ontology (GO) pathway enrichment analysis of DEGs in TCGA-STAD cohort; (C-D) KEGG and GO pathway enrichment analysis of DEGs in GEO cohorts.

Figure S3. The PrognosisScore’s prognostic significance. Survival impact of the PrognosisScore, Kaplan-Meier curves for overall survival (OS) of different pTNM stage in the TCGA-STAD cohort, Pathologic_T:Tumor, Pathologic_N:Node, Pathologic_M:Metastasis.

Figure S4. The boxplot and Kaplan-Meier curves of ATP4A, ALDH3A1, and BGN in TCGA-STAD.
